# Broad-Spectrum Antibiotic Use at the End of Life in Patients With Advanced Cancer

**DOI:** 10.1001/jamanetworkopen.2025.30980

**Published:** 2025-09-09

**Authors:** Jeong-Han Kim, Jiwon Yu, Shin Hye Yoo, Jin-Ah Sim, Bhumsuk Keam, Dae Seog Heo

**Affiliations:** 1 Division of Infectious Diseases, Department of Internal Medicine, Ewha Woman University College of Medicine, Mokdong Hospital, Seoul, South Korea; 2 Department of Artificial Intelligence Convergence, Hallym University, Chuncheon, South Korea; 3 Department of Biomedical Sciences, Seoul National University College of Medicine, Seoul, South Korea; 4 Center for Palliative Care and Clinical Ethics, Seoul National University Hospital, Seoul, South Korea; 5 Department of Human Systems Medicine, Seoul National University College of Medicine, Seoul, South Korea; 6 Department of Internal Medicine, Hallym University Dongtan Sacred Heart Hospital, Hallym University College of Medicine, Hwaseong, Gyeonggi, South Korea; 7 Department of Internal Medicine, Seoul National University Hospital, Seoul National University College of Medicine, Seoul, South Korea; 8 Patient-Centered Clinical Research Coordinating Center, National Evidence-Based Healthcare Collaborating Agency, Seoul, South Korea

## Abstract

**Importance:**

Patients with advanced cancer frequently receive broad-spectrum antibiotics, but changing use patterns across the end-of-life trajectory remain poorly understood.

**Objective:**

To describe the patterns of broad-spectrum antibiotic use across defined end-of-life intervals in patients with advanced cancer.

**Design, Setting, and Participants:**

This nationwide, population-based, retrospective cohort study used data from the South Korean National Health Insurance Service database to examine broad-spectrum antibiotic use among patients with advanced cancer who died between July 1, 2002, and December 31, 2021. Data extraction and analysis were conducted between September 2023 and August 2024.

**Exposure:**

A diagnosis of lung cancer, liver cancer, stomach cancer, colorectal cancer, pancreatic cancer, prostate cancer, gallbladder and biliary tract cancer, breast cancer, non-Hodgkin lymphoma, leukemia, or multiple myeloma.

**Main Outcomes and Measures:**

The use of broad-spectrum antibiotics (ie, antipseudomonal β-lactams, carbapenems, or glycopeptides) was evaluated using 2 metrics according to end-of-life trajectory: (1) prescription proportion (percentage of patients receiving antibiotics) and (2) consumption amount (days of therapy per 1000 patient-days). The end-of-life trajectory was divided into 5 intervals: T1 (6 months to 3 months before death), T2 (3 months to 1 month before death), T3 (1 month to 2 weeks before death), T4 (2 weeks to 1 week before death), and T5 (final week before death). Logistic regression was performed to calculate odds ratios and 95% CIs for antibiotic prescription proportion without adjustment for multiple comparisons, and Poisson regression was used to calculate adjusted relative risks.

**Results:**

Among the 515 366 decedents included, the mean (SD) age was 68.8 (11.7) years, and 347 327 (67.4%) were male. A total of 483 405 patients (93.8%) had solid tumors, with lung cancer (122 142 patients [23.7%]) being the most common type. Overall, 288 151 patients (55.9%) received broad-spectrum antibiotics during the last 6 months of life. The proportion of patients receiving broad-spectrum antibiotics peaked during T2, with 144 920 (28.1%) receiving at least 1 dose, and declined to 68 564 (13.3%) during T5. In contrast, total consumption peaked during T3, reaching 190.0 days of therapy per 1000 patient-days. These patterns were consistent across antibiotic classes and cancer types. During the last week of life, patients with leukemia had the highest exposure to broad-spectrum antibiotics compared with those with lung cancer, both for prescription proportions (crude odds ratio, 1.50; 95% CI, 1.43-1.58) and total consumption (adjusted relative risk, 1.21; 95% CI, 1.19-1.23).

**Conclusions and Relevance:**

In this cohort study of patients with advanced cancer, broad-spectrum antibiotic use increased from 3 months to 2 weeks before death, suggesting that this may be a key period for optimizing use and aligning care with patient goals.

## Key Points

**Question:**

What are the patterns of broad-spectrum antibiotic use at the end of life among patients with advanced cancer?

**Findings:**

In this nationwide cohort study including 515 366 decedents with advanced cancer, the proportion of patients receiving broad-spectrum antibiotics peaked at 3 months to 1 month before death, whereas total antibiotic consumption was highest at 1 month to 2 weeks before death. Broad-spectrum antibiotic exposure was consistently more frequent in hematologic cancers than in solid tumors across all periods.

**Meaning:**

These findings suggest that broad-spectrum antibiotic use in patients with advanced cancer was not randomly distributed but clustered in specific end-of-life periods, and this knowledge could be used to optimize use and align care with patient goals.

## Teaser

This cohort study examines the patterns of broad-spectrum antibiotic use across defined end-of-life intervals in patients with advanced cancer.

## Introduction

Patients with advanced cancer frequently receive antibiotics owing to a heightened risk of bacterial infections, which arises from functional decline, malnutrition, compromised host defenses, immunosuppression related to the disease and its treatment, and the presence of various vascular and drainage catheters.^1,2^ Physicians often prescribe antibiotics to alleviate inflammatory symptoms, such as fever, and as a temporary intervention to manage infections associated with advanced cancer in an attempt to prolong life.^1,3^ However, excessive antibiotic use in these patients may compromise the quality of end-of-life care by causing adverse effects, such as nausea, diarrhea, and *Clostridioides difficile* infection.^4,5^ In addition, intravenous administration of antibiotics may complicate the transition to a comfortable home environment during the dying process.^6,7^ Moreover, the overuse of broad-spectrum antibiotics contributes to the emergence of multidrug-resistant organisms, posing a major public health concern.^8^

Given these concerns, careful consideration of antibiotic prescribing and use is essential in this population. However, data on how broad-spectrum antibiotics are used across the end-of-life trajectory in patients with advanced cancer remain limited, making it difficult to establish appropriate clinical guidance. Understanding patterns of broad-spectrum antibiotics throughout the end-of-life trajectory could guide the development of antimicrobial stewardship protocols aimed at minimizing the adverse outcomes of excessive prescribing while ensuring alignment with patient goal-concordant care.

Functional decline in patients with advanced cancer follows a predictable trajectory, with a prolonged period of relative stability followed by a more-rapid deterioration in the last weeks of life.^9,10^ This progression is usually associated with an increased symptom burden and frequent hospitalizations, which, in turn, drives antibiotic use.^11,12^ Given the progressive nature of advanced cancer, it is important to characterize how broad-spectrum antibiotic use varies across the different phases of the disease trajectory. Therefore, this study aimed to analyze patterns of broad-spectrum antibiotic use in patients with advanced cancer using a nationwide database.

## Methods

### Data Source

This nationwide, population-based, retrospective cohort study used the National Health Insurance (NHIS) database of South Korea. The NHIS, a single-payer system managed by the South Korean government, provides mandatory universal health insurance coverage to 97% of the population.^13^ The NHIS database systematically collects all claims for insured medical services, including demographic information, diagnostic codes based on the *International Statistical Classification of Diseases and Related Health Problems, Tenth Revision (ICD-10)*, prescription drug records, and death certificates for all enrolled citizens.^14^ In South Korea, antibiotics are strictly prescription-based, and all antibiotic utilization data are recorded in the NHIS database. For patients with cancer, the NHIS provides additional insurance coverage, requiring the assignment of a specific insurance code (V193) upon diagnosis confirmation. This code is activated at the time of cancer diagnosis, is updated periodically for patients receiving active treatment or follow-up care, and is deactivated 5 years after diagnosis for those considered successfully treated.

### Ethical Statement

This study was approved by the institutional review board of Seoul National University Hospital. The requirement for informed consent was waived because of the use of anonymized retrospective data, which were routinely collected as part of the health insurance process and linked to mortality data from the Korea National Statistical Office. This study followed the Strengthening the Reporting of Observational Studies in Epidemiology (STROBE) reporting guidelines for cohort studies.

### Study Population

We initially screened adult patients aged 18 years or older who had used health care services associated with a primary cancer diagnosis from 2002 to 2021. Eligible cancer types included lung, liver, stomach, colorectal, pancreatic, prostate, gallbladder and biliary tract, breast, non-Hodgkin lymphoma, leukemia, and multiple myeloma. A full list of corresponding *ICD-10* codes is provided in eTable 1 in Supplement 1. From this cohort, we included decedents whose specific cancer insurance code (V193) was maintained from 1 year before death until the time of death. The included study population was further classified by cancer type, considering only the primary diagnosis in patients with multiple cancer types.

### Study Outcome

We extracted antibiotic prescription data according to the World Health Organization’s Anatomical Therapeutic Chemical classification system, specifically focusing on systemic antibacterial agents categorized under the Anatomical Therapeutic Chemical J01 code.^15^ Among these, the broad-spectrum antibiotics of interest in this study included antipseudomonal β-lactam agents (penicillins and cephalosporins), carbapenems, and glycopeptides (eTable 2 in Supplement 1).

The duration of the end-of-life phase has been variably defined in the literature, ranging from 6 months before death to the last week.^16^ Accordingly, we categorized the end-of-life phase into the following periods: T1 (6 months to 3 months before death), T2 (3 months to 1 month before death), T3 (1 month to 2 weeks before death), T4 (2 weeks to 1 week before death), and T5 (1 week before death until death). These nonuniform time intervals were chosen to reflect the accelerating decline in clinical status that is commonly observed in patients with advanced cancer, particularly during the final month of life.^9^ The analysis was conducted using 2 approaches: first, by assessing changes in antibiotic use across each period, and second, by comparing these changes among cancer types, using lung cancer, the most frequent primary cancer among decedents, as the reference.^17^

Antibiotic use was assessed using 2 complementary measures: the proportion of patients who received antibiotics (prescription proportion) and the total amount of antibiotics administered (consumption amount). The prescription proportion was defined as the percentage of inpatients and outpatients who received at least 1 dose of antibiotics among all decedents. Antibiotic consumption was measured as days of therapy (DOT) per 1000 patient-days. DOT was defined as the aggregate number of days each patient received a specific broad-spectrum antibiotic, and the denominator reflected the total number of patient-days of observation accumulated by all patients during the assessment period, including both inpatient and outpatient days.^18^ The composite outcome was defined according to both measures: the prescription proportion of patients receiving at least 1 class of broad-spectrum antibiotics (antipseudomonal β-lactams, carbapenems, or glycopeptides) during the specified period, and the corresponding total DOTs per 1000 patient-days for those antibiotics.

### Statistical Analysis

Data extraction and analysis were conducted between September 2023 and August 2024. Continuous variables are expressed as mean and SD, whereas categorical variables are expressed as frequencies. Logistic regression was performed to calculate odds ratios (ORs) and 95% CIs for antibiotic prescription proportion without adjustment for multiple comparisons. Although individual-level covariates such as age, sex, and year of death were available, they were not included in the analysis because the outcome was defined as the proportion of antibiotic prescriptions in each time period (T1-T5) relative to the total number of prescriptions over the entire observation period. This aggregation made covariate adjustment methodologically infeasible. For antibiotic consumption amount, Poisson regression was used to calculate adjusted relative risks (aRR) and 95% CI, adjusting for the following variables in the base multivariable model: age, sex, household income status, and Charlson Comorbidity Index score. All analyses, including OR, aRR, proportions, and DOT for each of the time intervals (T1-T5), were calculated using the total cancer decedent cohort as the reference denominator. No missing variables were present in the regression analyses, because patients with missing baseline information were excluded. All statistical analyses were conducted using SAS statistical software version 9.4 (SAS Institute), and 2-sided *P* < .05 was considered statistically significant.

## Results

### Baseline Characteristics of the Study Population

We identified 515 366 decedents with cancer between 2002 and 2021. The mean (SD) age was 68.8 (11.7) years, and 347 327 patients (67.4%) were male (Table 1). A total of 377 254 patients (73.2%) had at least 1 noncancer chronic condition in addition to cancer. Overall, solid tumors accounted for the majority of primary cancers (483 405 patients [93.8%]), with lung cancer being the most common (122 141 patients [23.7%]), followed by liver cancer (85 283 patients [16.6%]) and stomach cancer (84 325 patients [16.4%]).

**Table 1.** Baseline Characteristics of the Study Population

Characteristics	Participants, No. (%)			*P* value
	All (N = 515 366)	Broad-spectrum antibiotics nonuser (n = 227 215)	Broad-spectrum antibiotics user (n = 288 151)	
Age, mean (SD), y	68.8 (11.7)	69.8 (11.7)	68.0 (11.7)	<.001
Sex				
Male	347 327 (67.4)	151 432 (66.6)	195 895 (67.9)	<.001
Female	168 039 (32.6)	75 783 (33.4)	92 256 (32.1)	
Household income				
First quartile (lowest)	106 995 (20.8)	48 270 (21.3)	58 725 (20.4)	<.001
Second quartile	59 142 (11.5)	26 682 (11.7)	32 460 (11.3)	
Third quartile	78 394 (15.2)	34 808 (15.3)	43 586 (15.1)	
Fourth quartile	106 434 (20.7)	46 497 (20.5)	59 937 (20.8)	
Fifth quartile (highest)	164 401 (31.8)	70 958 (31.2)	93 443 (32.4)	
Charlson Comorbidity Index score^a^				
0-1	139 470 (27.0)	57 974 (25.5)	81 496 (28.3)	<.001
2-3	141 012 (27.4)	64 349 (28.3)	76 663 (26.6)	
≥ 4	234 884 (45.6)	104 892 (46.2)	129 992 (45.1)	
Cancer type				
Lung	122 141 (23.7)	46 103 (20.3)	76 038 (26.4)	<.001
Liver	85 283 (16.6)	43 517 (19.2)	41 766 (14.5)	
Stomach	84 325 (16.4)	42 779 (18.8)	41 546 (14.4)	
Colorectal	77 132 (15.0)	39 570 (17.4)	37 562 (13.0)	
Pancreatic	32 716 (6.4)	14 035 (6.2)	18 681 (6.5)	
Prostate	31 570 (6.1)	15 020 (6.6)	16 550 (5.7)	
Gallbladder and biliary tract	28 061 (5.4)	10 435 (4.6)	17 626 (6.1)	
Breast	22 177 (4.3)	9795 (4.3)	12 382 (4.3)	
Non-Hodgkin lymphoma	13 105 (2.5)	2834 (1.2)	10 271 (3.6)	
Leukemia	10 777 (2.0)	1263 (0.6)	9514 (3.3)	
Multiple myeloma	8079 (1.6)	1864 (0.8)	6215 (2.2)	

^a^ Excludes cancer status.

Most patients (466 328 patients [90.5%]) received at least 1 systemic antibiotic during the last 6 months of life, and 288 151 patients (55.9%) received broad-spectrum antibiotics. The demographic characteristics of patients who received broad-spectrum antibiotics were generally similar to those who did not. However, compared with the nonuse group, patients in the broad-spectrum antibiotic use group tended to be younger, were more likely to be male, and had lower noncancer Charlson Comorbidity Index scores. Also, the broad-spectrum antibiotic use group had a higher proportion of patients with lung cancer and hematologic cancers than the nonuse group.

### Patterns of Antibiotics Prescription Proportions

**Figure 1.**
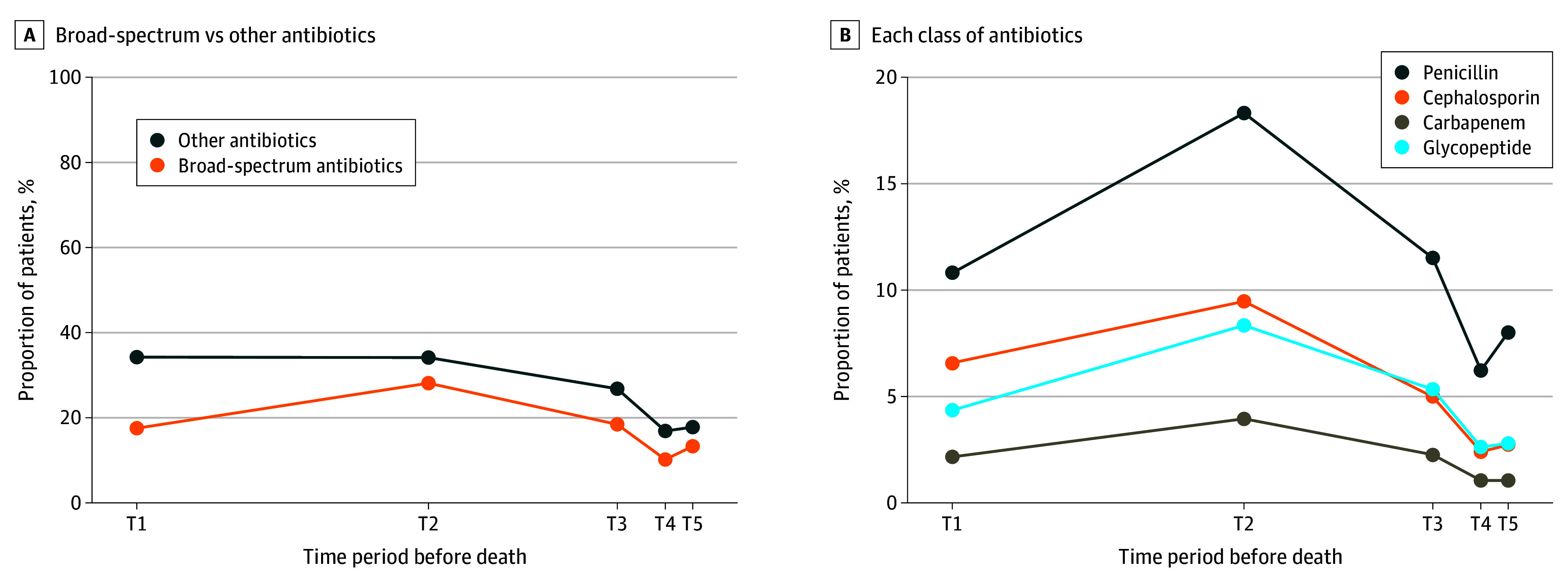
Proportion of Patients Receiving Antibiotics During the Last 6 Months of Life A, Proportion of patients receiving broad-spectrum and other antibiotics across time periods, defined as T1 (6 to 3 months before death), T2 (3 months to 1 month before death), T3 (1 month to 2 weeks before death), T4 (2 weeks to 1 week before death), and T5 (final week before death). B, Proportion of patients receiving each class of broad-spectrum antibiotics across time periods.

During the last 6 months of life, the proportion of patients receiving broad-spectrum antibiotics was highest in the T2 period (144 920 patients [28.1%]), followed by a gradual decline to the T5 period (68 564 patients [13.3%]) (Figure 1A). A similar pattern was observed across all classes of broad-spectrum antibiotics (Figure 1B). In the T2 period, the most frequently prescribed class was antipseudomonal penicillins (94 417 patients [18.3%]), followed by antipseudomonal cephalosporins (48 830 patients [9.5%]), glycopeptides (43 000 patients [8.3%]), and carbapenems (20 333 patients [3.9%]).

Cancer type–specific likelihoods of broad-spectrum antibiotic exposure across time intervals are summarized in Table 2 and eFigure 1 in Supplement 1. Compared with lung cancer as the reference, patients with gallbladder and biliary tract cancer had a slightly higher likelihood of receiving broad-spectrum antibiotics during the T1 to T2 period (crude OR at T1, 1.42 [95% CI, 1.38-1.46]; crude OR at T2, 1.33 [95% CI, 1.30-1.37]). Patients with hematologic cancers (non-Hodgkin lymphoma, leukemia, and multiple myeloma) showed consistently higher odds across all periods (eg, crude ORs for leukemia of 3.92 [95% CI, 3.77-4.08] at T1 and 1.50 [95% CI, 1.43-1.58] at T5). In contrast, patients with other solid tumors had consistently lower odds of receiving broad-spectrum antibiotics than those with lung cancer from T3 to T5.

**Table 2.** Relative Likelihood of Prescription Proportions by Cancer Type and Time Before Death

Cancer type	T1 (6 to 3 mo before death)		T2 (3 to 1 mo before death)		T3 (1 mo to 2 wk before death)		T4 (2 to 1 wk before death)		T5 (final week before death)	
	OR (95% CI)^a^	*P* value	OR (95% CI)^a^	*P* value	OR (95% CI)^a^	*P* value	OR (95% CI)^a^	*P* value	OR (95% CI)^a^	*P* value
Lung	1 [Reference]	NA	1 [Reference]	NA	1 [Reference]	NA	1 [Reference]	NA	1 [Reference]	NA
Liver	0.75 (0.74-0.77)	<.001	0.75 (0.74-0.77)	<.001	0.72 (0.70-0.74)	<.001	0.72 (0.70-0.74)	<.001	0.60 (0.58-0.61)	<.001
Stomach	0.70 (0.69-0.72)	<.001	0.77 (0.76-0.79)	<.001	0.73 (0.71-0.74)	<.001	0.70 (0.68-0.72)	<.001	0.63 (0.61-0.64)	<.001
Colorectal	0.72 (0.70-0.74)	<.001	0.73 (0.71-0.74)	<.001	0.70 (0.69-0.72)	<.001	0.70 (0.68-0.72)	<.001	0.65 (0.63-0.67)	<.001
Pancreatic	0.99 (0.96-1.02)	.54	1.01 (0.99-1.04)	.41	0.92 (0.89-0.94)	<.001	0.86 (0.83-0.89)	<.001	0.66 (0.63-0.68)	<.001
Prostate	0.73 (0.71-0.76)	<.001	0.77 (0.75-0.79)	<.001	0.77 (0.75-0.80)	<.001	0.85 (0.81-0.88)	<.001	0.86 (0.83-0.89)	<.001
Gallbladder and biliary tract	1.42 (1.38-1.46)	<.001	1.33 (1.30-1.37)	<.001	1.05 (1.01-1.08)	.005	0.88 (0.85-0.99)	.004	0.72 (0.69-0.75)	<.001
Breast	0.83 (0.80-0.87)	<.001	0.86 (0.83-0.88)	<.001	0.85 (0.81-0.88)	<.001	0.88 (0.84-0.92)	.005	0.80 (0.77-0.83)	.001
Non-Hodgkin lymphoma	2.53 (2.43-2.63)	<.001	2.23 (2.15-2.31)	<.001	1.59 (1.53-1.65)	<.001	1.35 (1.29-1.42)	<.001	1.14 (1.09-1.19)	<.001
Leukemia	3.92 (3.77-4.08)	<.001	3.22 (3.09-3.35)	<.001	2.04 (1.96-2.13)	<.001	1.55 (1.47-1.64)	<.001	1.50 (1.43-1.58)	<.001
Multiple myeloma	1.85 (1.76-1.95)	<.001	1.72 (1.64-1.80)	<.001	1.43 (1.36-1.50)	<.001	1.26 (1.18-1.34)	<.001	1.27 (1.20-1.34)	<.001

Abbreviations: NA, not applicable; OR, odds ratio.

^a^ Crude ORs were calculated by logistic regression analysis.

### Patterns of Antibiotics Consumption Amount

**Figure 2.**
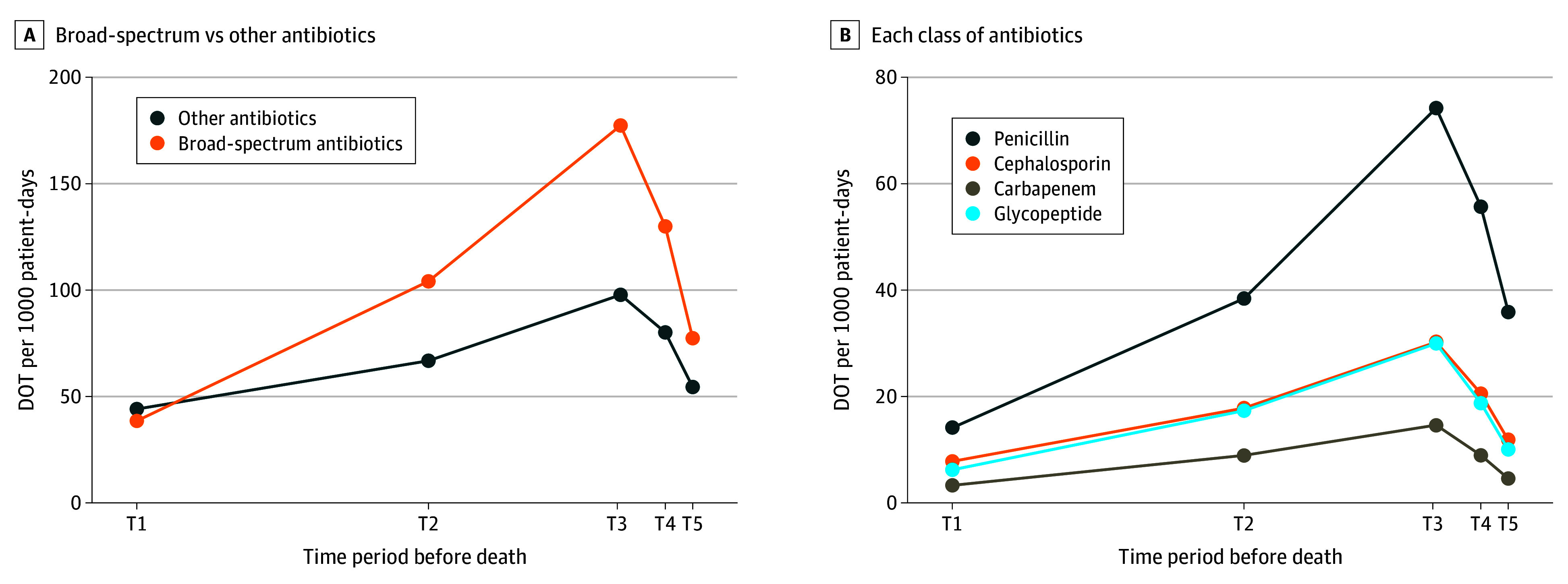
Antibiotic Consumption Patient-Days During the Last 6 Months of Life A, Days of therapy (DOT) per 1000 patient-days for broad-spectrum and other antibiotics across time periods, defined as T1 (6 to 3 months before death), T2 (3 months to 1 month before death), T3 (1 month to 2 weeks before death), T4 (2 weeks to 1 week before death), and T5 (final week before death). B, DOT per 1000 patient-days by class of broad-spectrum antibiotics across time periods.

Regarding broad-spectrum antibiotic consumption amount, DOTs peaked during the T3 period at 190.0 DOTs per 1000 patient-days, followed by a gradual decline to 77.4 DOTs per 1000 patient-days (Figure 2A). A similar pattern was observed for other antibiotic types and individual classes of broad-spectrum antibiotics (Figure 2B). During the T3 period, the most frequently administered class was antipseudomonal penicillins (74.2 DOTs per 1000 patient-days), followed by glycopeptides (30.3 DOTs per 1000 patient-days), antipseudomonal cephalosporins (30.1 DOTs per 1000 patient-days), and carbapenems (14.6 DOTs per 1000 patient-days).

Cancer type–specific relative antibiotic consumption across time intervals is summarized in Table 3 and eFigure 2 in Supplement 1. Compared with lung cancer as the reference, the relative antibiotic consumption during the T1 to T2 period varied across solid tumors without a consistent directional pattern. However, from T3 to T5, the relative antibiotic consumption was lower across all solid cancer types. In contrast, hematologic cancers exhibited consistently higher antibiotic consumption across all periods, with the most pronounced differences observed in patients with leukemia (aRR at T1, 2.14 [95% CI, 2.12-2.15]; aRR at T5, 1.21 [95% CI, 1.19-1.23]).

**Table 3.** Relative Antibiotics Consumptions by Cancer Type and Time Before Death

Cancer type	T1 (6 to 3 mo before death)		T2 (3 to 1 mo before death)		T3 (1 mo to 2 wk before death)		T4 (2 to 1 wk before death)		T5 (final week before death)	
	aRR (95% CI)^a^	*P* value	aRR (95% CI)^a^	*P* value	aRR (95% CI)^a^	*P* value	aRR (95% CI)^a^	*P* value	aRR (95% CI)^a^	*P* value
Lung	1 [Reference]	NA	1 [Reference]	NA	1 [Reference]	NA	1 [Reference]	NA	1 [Reference]	NA
Liver	1.01 (1.01-1.02)	<.001	0.98 (0.98-0.99)	<.001	0.91 (0.90-0.91)	<.001	0.88 (0.87-0.89)	<.001	0.91 (0.90-0.91)	<.001
Stomach	1.05 (1.05-1.06)	<.001	1.02 (1.02-1.03)	<.001	0.96 (0.95-0.96)	<.001	0.93 (0.92-0.94)	<.001	0.94 (0.92-0.95)	<.001
Colorectal	1.09 (1.08-1.09)	<.001	1.02 (1.01-1.02)	<.001	0.94 (0.94-0.95)	<.001	0.94 (0.93-0.95)	<.001	0.96 (0.94-0.97)	<.001
Pancreatic	0.94 (0.93-0.94)	<.001	0.99 (0.98-0.99)	<.001	0.94 (0.93-0.95)	<.001	0.92 (0.90-0.93)	<.001	0.94 (0.93-0.96)	<.001
Prostate	1.19 (1.19-1.20)	<.001	1.07 (1.06-1.08)	<.001	0.98 (0.97-0.98)	<.001	0.98 (0.97-0.99)	.01	0.96 (0.94-0.98)	<.001
Gallbladder and biliary tract	1.13 (1.12-1.14)	<.001	1.12 (1.12-1.13)	<.001	0.97 (0.96-0.98)	<.001	0.96 (0.95-0.98)	<.001	0.97 (0.96-0.99)	<.001
Breast	0.98 (0.97-0.98)	<.001	0.97 (0.97-0.98)	<.001	0.94 (0.93-0.95)	<.001	0.96 (0.94-0.97)	<.001	0.97 (0.95-0.99)	.003
Non-Hodgkin lymphoma	1.44 (1.43-1.45)	<.001	1.39 (1.38-1.39)	<.001	1.23 (1.21-1.23)	<.001	1.14 (1.13-1.16)	<.001	1.13 (1.11-1.15)	.01
Leukemia	2.14 (2.12-2.15)	<.001	1.88 (1.86-1.88)	<.001	1.47 (1.45-1.48)	<.001	1.35 (1.34-1.38)	<.001	1.21 (1.19-1.23)	<.001
Multiple myeloma	1.35 (1.34-1.36)	<.001	1.33 (1.32-1.34)	<.001	1.22 (1.21-1.23)	<.001	1.13 (1.11-1.15)	<.001	1.13 (1.11-1.15)	<.001

Abbreviations: aRR, adjusted relative risk; NA, not applicable.

^a^ aRRs were calculated by Poisson regression analysis adjusted for age, sex, household income, and Charlson Comorbidity Index score.

## Discussion

Using nationwide data from South Korea, this large cohort study examined broad-spectrum antibiotic prescribing patterns during the last 6 months of life in patients with advanced cancer, focusing on the proportion of patients receiving antibiotics and the overall antibiotic consumption amount represented as DOT. Understanding these patterns is essential, as perceptions of the definition and timing of the end-of-life trajectory in patients with advanced cancer can vary across countries, cultures, and even among physicians.^16^ In this population, decisions regarding broad-spectrum antibiotic use are particularly complex, requiring careful consideration of the clinical factors and the emotional and ethical aspects.^19^ By identifying patterns in broad-spectrum antibiotic use across different phases of the end-of-life trajectory, our study provides critical insights to help optimize antimicrobial stewardship strategies in this vulnerable population.

The increase in the proportion of patients receiving broad-spectrum antibiotics during the T2 period and the peak in overall consumption during T3 may indicate the occurrence of a common turning point among patients with advanced cancer between 3 months and 2 weeks before death. These antibiotic use patterns were consistent across solid and hematologic cancers, as well as among different types of solid tumors and across various classes of broad-spectrum antibiotics. This turning point may be related to a general decline in functional status that typically begins around 3 months before death in patients with advanced cancer.^20,21^ This functional deterioration can lead to more-frequent hospitalizations and prolonged antibiotic treatment. In addition, disease progression may induce a refractory inflammatory state, further contributing to the increased use of broad-spectrum antibiotics.^22^ Although our study did not assess performance status using standardized tools such as the Eastern Cooperative Oncology Group or Karnofsky scale, nor did it evaluate markers of disease progression directly, a study by Marra et al^23^ reported a sharp increase in antibiotic use at the time of hospice transfer. Although their study included patients dying from various chronic conditions, this trend is consistent with the patterns observed in our cohort of patients with advanced cancer.

However, the reason for the discrepancy between the peak timing of antibiotic prescription proportions and total consumption amount remains unclear. One possible explanation is methodological: because prescription proportion reflects whether a patient received at least 1 dose during a given period, longer intervals such as T2 (3 months to 1 month before death) naturally increase the likelihood of capturing any use. In contrast, shorter intervals like T3 (1 month to 2 weeks) may yield lower proportions, despite higher intensity. This time window association may partly explain the higher prescription proportion in T2. In addition, the peak in antibiotic consumption at T3 likely reflects a shift in care intensity. Although fewer patients receive antibiotics during this period, those who do are often hospitalized and receive prolonged courses, suggesting escalating clinical deterioration and more-intensive care.^24^

Exposure of broad-spectrum antibiotic use varied across cancer types compared with lung cancer, as demonstrated by the divergence of both prescription proportions and total consumption amount. These variations may suggest that even among patients with advanced cancer, the progression of illness and corresponding clinical responses can differ meaningfully by cancer type. For instance, the higher likelihood of prescription observed in gall bladder and biliary tract cancer during the T1 to T2 period may reflect earlier clinical deterioration or more proactive infection control. These cancers often lead to obstructive cholangitis due to mass effect, which may prompt earlier or more frequent antibiotic use, even in the earlier stages of the end-of-life trajectory. Although the lower proportions and consumption observed in solid tumors during the T3 to T5 period may indicate a more conservative approach or earlier transition to comfort-oriented care, hematologic cancers exhibited persistently high antibiotic consumption, likely reflecting the greater degree of immunosuppression and continued need for infection management even in the final stages of life.^25^

The emergence of untreatable multidrug-resistant organisms is an urgent global public health concern, and antimicrobial stewardship remains one of the most effective strategies to address this issue.^26^ Our study may offer important insights for stewardship efforts targeting patients with advanced cancer in the end-of-life phase, suggesting a potential turning point when more deliberate prescribing decisions could be considered. Although these findings highlight a window for targeted stewardship interventions, we acknowledge that accurately predicting death within specific time frames, such as 3 or 6 months, remains difficult in clinical practice. Therefore, time-based approaches could be applied with flexibility and clinical judgment.^27^ Time-limited antibiotic trials, initiating treatment with planned reassessment, offer a practical strategy to align use with patient goals and clinical context across the disease course. Infectious disease physicians may play an essential role in engaging patients in goals-of-care discussions to ensure that antibiotic treatments align with the prognosis, goals, and priorities of patients who experience an incurable inflammatory course.^19^

Another important consideration alongside antimicrobial stewardship in this patient population is palliative care, which can help align medical decisions with the values and goals of the patient. Early palliative care has been shown in various studies to reduce the use of aggressive treatments and the length of hospitalization.^28,29,30^ Our previous single-center study conducted in an acute care hospital demonstrated that receiving in-hospital palliative care consultation was associated with significantly lower broad-spectrum antibiotic use, particularly carbapenems.^12,31^ Also, patients who received palliative care consultation may have been more likely to transition to hospice facilities rather than remaining in acute care hospitals at the end of life, thereby reducing the likelihood of intensive antibiotic treatment. Rather than emphasizing aggressive broad-spectrum antibiotic treatment, palliative and hospice care prioritize minimizing suffering and maximizing the quality of life.

### Limitations

Our study has certain limitations. First, although this study included decedents with a documented cancer diagnosis and sustained cancer-specific insurance status (V193) until death, we could not confirm that all deaths were directly attributable to cancer progression. Some patients may have died from other chronic conditions or unrelated causes such as injuries or acute medical events, particularly in the context of multimorbidity. Second, our study was based on the NHIS claims database, which has inherent limitations in accurately capturing clinical diagnoses. The use of *ICD-10* codes for identifying cancer types and associated conditions may lead to potential misclassification or coding errors. Third, since only extractable baseline characteristics were included in the adjustments, this analysis was limited by the inability to account for unmeasured confounders, such as disease severity, care setting transitions over time, complex patient-related medical conditions, and microbiologic data, all of which may affect antibiotic prescribing patterns. Whether antibiotic use was intended to treat a confirmed infection to prolong life or empirically to relieve infection-mediated or cancer-mediated inflammation-induced symptoms was unclear. Therefore, we are unable to provide recommendations on the extent to which broad-spectrum antibiotic use should be reduced. Fourth, this study was conducted using data from South Korea, and the findings may not be broadly applicable to other health care systems or global populations.

## Conclusions

Our large cohort study provides substantial evidence that patients with cancer at the end of life are exposed to high levels of broad-spectrum antibiotic treatment from 3 months to 2 weeks before death. Future studies are needed to identify optimal targets during the end-of-life period for achieving synergy between antimicrobial stewardship programs and hospice or palliative care teams.

## Notes

Supplement 1. **eTable 1.** *ICD-10* Codes Used to Identify Eligible Cancer Types in the Study **eTable 2.** The Broad-Spectrum Antibiotics Lists **eFigure 1.** Proportion of Broad-Spectrum Antibiotic Use by Time Period **eFigure 2.** Days of Therapy per 1000 Patient-Days of Broad-Spectrum Antibiotic Use by Time Period

Supplement 2.
Data Sharing Statement

## References

[1] Macedo F, Nunes C, Ladeira K, . Antimicrobial therapy in palliative care: an overview. Support Care Cancer. 2018;26(5):1361-1367. doi:10.1007/s00520-018-4090-8 29435712

[2] Rolston KV. Infections in cancer patients with solid tumors: a review. Infect Dis Ther. 2017;6(1):69-83. doi:10.1007/s40121-017-0146-1 28160269 PMC5336421

[3] Thompson AJ, Silveira MJ, Vitale CA, Malani PN. Antimicrobial use at the end of life among hospitalized patients with advanced cancer. Am J Hosp Palliat Care. 2012;29(8):599-603. doi:10.1177/1049909111432625 22218916

[4] Delgado A, Reveles IA, Cabello FT, Reveles KR. Poorer outcomes among cancer patients diagnosed with *Clostridium difficile* infections in United States community hospitals. BMC Infect Dis. 2017;17(1):448. doi:10.1186/s12879-017-2553-z 28645266 PMC5481960

[5] Wiström J, Norrby SR, Myhre EB, . Frequency of antibiotic-associated diarrhoea in 2462 antibiotic-treated hospitalized patients: a prospective study. J Antimicrob Chemother. 2001;47(1):43-50. doi:10.1093/jac/47.1.43 11152430

[6] Datta R, Zhu M, Han L, Allore H, Quagliarello V, Juthani-Mehta M. Increased length of stay associated with antibiotic use in older adults with advanced cancer transitioned to comfort measures. Am J Hosp Palliat Care. 2020;37(1):27-33. doi:10.1177/1049909119855617 31185722 PMC6868290

[7] Merel SE, Meier CA, McKinney CM, Pottinger PS. Antimicrobial use in patients on a comfort care protocol: a retrospective cohort study. J Palliat Med. 2016;19(11):1210-1214. doi:10.1089/jpm.2016.0094 27309999 PMC5105343

[8] Levin PD, Simor AE, Moses AE, Sprung CL. End-of-life treatment and bacterial antibiotic resistance: a potential association. Chest. 2010;138(3):588-594. doi:10.1378/chest.09-2757 20472860

[9] Amblàs-Novellas J, Murray SA, Espaulella J, . Identifying patients with advanced chronic conditions for a progressive palliative care approach: a cross-sectional study of prognostic indicators related to end-of-life trajectories. BMJ Open. 2016;6(9):e012340. doi:10.1136/bmjopen-2016-012340 27645556 PMC5030552

[10] Jordhøy MS, Fayers P, Loge JH, Saltnes T, Ahlner-Elmqvist M, Kaasa S. Quality of life in advanced cancer patients: the impact of sociodemographic and medical characteristics. Br J Cancer. 2001;85(10):1478-1485. doi:10.1054/bjoc.2001.2116 11720432 PMC2363932

[11] Mercadante S, Valle A, Sabba S, . Pattern and characteristics of advanced cancer patients admitted to hospices in Italy. Support Care Cancer. 2013;21(4):935-939. doi:10.1007/s00520-012-1608-3 23052914

[12] Kim JH, Yoo SH, Keam B, Heo DS. The impact of palliative care consultation on reducing antibiotic overuse in hospitalized patients with terminal cancer at the end of life: a propensity score-weighting study. J Antimicrob Chemother. 2022;78(1):302-308. doi:10.1093/jac/dkac405 36424671

[13] Kim DH, Han K, Kim SW. Effects of antibiotics on the development of asthma and other allergic diseases in children and adolescents. Allergy Asthma Immunol Res. 2018;10(5):457-465. doi:10.4168/aair.2018.10.5.457 30088366 PMC6082825

[14] Cheol Seong S, Kim YY, Khang YH, . Data resource profile: the National Health Information Database of the National Health Insurance Service in South Korea. Int J Epidemiol. 2017;46(3):799-800. doi:10.1093/ije/dyw253 27794523 PMC5837262

[15] WHO Collaborating Centre for Drug Statistics Methodology. ATC classification index with DDDs. 2022. Accessed August 4, 2025. https://atcddd.fhi.no/atc_ddd_index_and_guidelines/atc_ddd_index/

[16] Hui D, Nooruddin Z, Didwaniya N, . Concepts and definitions for “actively dying,” “end of life,” “terminally ill,” “terminal care,” and “transition of care”: a systematic review. J Pain Symptom Manage. 2014;47(1):77-89. doi:10.1016/j.jpainsymman.2013.02.021 23796586 PMC3870193

[17] Bray F, Laversanne M, Sung H, . Global cancer statistics 2022: GLOBOCAN estimates of incidence and mortality worldwide for 36 cancers in 185 countries. CA Cancer J Clin. 2024;74(3):229-263. doi:10.3322/caac.21834 38572751

[18] Polk RE, Fox C, Mahoney A, Letcavage J, MacDougall C. Measurement of adult antibacterial drug use in 130 US hospitals: comparison of defined daily dose and days of therapy. Clin Infect Dis. 2007;44(5):664-670. doi:10.1086/511640 17278056

[19] Karlin D, Pham C, Furukawa D, . State-of-the-art review: use of antimicrobials at the end of life. Clin Infect Dis. 2024;78(3):e27-e36. doi:10.1093/cid/ciad735 38301076

[20] Lunney JR, Lynn J, Foley DJ, Lipson S, Guralnik JM. Patterns of functional decline at the end of life. JAMA. 2003;289(18):2387-2392. doi:10.1001/jama.289.18.2387 12746362

[21] Morgan DD, Tieman JJ, Allingham SF, Ekström MP, Connolly A, Currow DC. The trajectory of functional decline over the last 4 months of life in a palliative care population: a prospective, consecutive cohort study. Palliat Med. 2019;33(6):693-703. doi:10.1177/0269216319839024 30916620

[22] Oh DY, Kim JH, Kim DW, . Antibiotic use during the last days of life in cancer patients. Eur J Cancer Care (Engl). 2006;15(1):74-79. doi:10.1111/j.1365-2354.2005.00603.x 16441680

[23] Marra AR, Clore GS, Balkenende E, . Association of entry into hospice or palliative care consultation during acute care hospitalization with subsequent antibiotic utilization. Clin Microbiol Infect. 2023;29(1):107.e1-107.37. doi:10.1016/j.cmi.2022.07.018 35931374

[24] Teno JM, Gozalo PL, Bynum JP, . Change in end-of-life care for Medicare beneficiaries: site of death, place of care, and health care transitions in 2000, 2005, and 2009. JAMA. 2013;309(5):470-477. doi:10.1001/jama.2012.207624 23385273 PMC3674823

[25] El-Jawahri A, Nelson AM, Gray TF, Lee SJ, LeBlanc TW. Palliative and end-of-life care for patients with hematologic malignancies. J Clin Oncol. 2020;38(9):944-953. doi:10.1200/JCO.18.02386 32023164 PMC8462532

[26] Barlam TF, Cosgrove SE, Abbo LM, . Implementing an antibiotic stewardship program: guidelines by the Infectious Diseases Society of America and the Society for Healthcare Epidemiology of America. Clin Infect Dis. 2016;62(10):e51-e77. doi:10.1093/cid/ciw118 27080992 PMC5006285

[27] Quill TE, Holloway R. Time-limited trials near the end of life. JAMA. 2011;306(13):1483-1484. doi:10.1001/jama.2011.1413 21972312

[28] Zaborowski N, Scheu A, Glowacki N, Lindell M, Battle-Miller K. Early palliative care consults reduce patients’ length of stay and overall hospital costs. Am J Hosp Palliat Care. 2022;39(11):1268-1273. doi:10.1177/10499091211067811 35061508 PMC9527348

[29] Ma J, Chi S, Buettner B, . Early palliative care consultation in the medical ICU: a cluster randomized crossover trial. Crit Care Med. 2019;47(12):1707-1715. doi:10.1097/CCM.0000000000004016 31609772 PMC6861688

[30] Yoo SH, Keam B, Kim M, Kim TM, Kim DW, Heo DS. The effect of hospice consultation on aggressive treatment of lung cancer. Cancer Res Treat. 2018;50(3):720-728. doi:10.4143/crt.2017.169 28707460 PMC6056966

[31] Kim JH, Yoo SH, Keam B, Heo DS. Antibiotic prescription patterns during last days of hospitalized patients with advanced cancer: the role of palliative care consultation. J Antimicrob Chemother. 2023;78(7):1694-1700. doi:10.1093/jac/dkad156 37220755

