# Supplementary material for: Broad-Spectrum Antibiotic Use at the End of Life in Patients With Advanced Cancer
Source: JAMA Netw Open. 2025 Sep 9;8(9):e2530980. doi: 10.1001/jamanetworkopen.2025.30980 (PMC12421337; doi:10.1001/jamanetworkopen.2025.30980)
Supplement: Supplement 1. — eTable 1. ICD-10 Codes Used to Identify Eligible Cancer Types in the Study eTable 2. The Broad-Spectrum Antibiotics Lists eFigure 1. Proportion of Broad-Spectrum Antibiotic Use by Time Period eFigure 2. Days of Therapy per 1000 Patient-Days of Broad-Spectrum Antibiotic Use by Time Period [file jamanetwopen-e2530980-s001.pdf]

Supplemental Online Content

Kim JH, Yu J, Yoo SH, Sim JA, Keam B, Heo DS. Broad-spectrum antibiotic use at the end of life in patients with advanced cancer. *JAMA Netw Open*. 2025;8(9):e2530980. doi:10.1001/jamanetworkopen.2025.30980

eTable 1. *ICD-10* Codes Used to Identify Eligible Cancer Types in the Study
eTable 2. The Broad-Spectrum Antibiotics Lists
eFigure 1. Proportion of Broad-Spectrum Antibiotic Use by Time Period
eFigure 2. Days of Therapy per 1000 Patient-Days of Broad-Spectrum Antibiotic Use by Time Period

This supplemental material has been provided by the authors to give readers additional information about their work.

© 2025 Kim JH et al. *JAMA Network Open*.

eTable 1. ***ICD-10*** **Codes Used to Identify Eligible Cancer Types in the Study**

Type	ICD-10 code
Lung cancer	C33, C34
Liver cancer	C22
Stomach cancer	C16
Colorectal cancer	C18–C21
Pancreatic cancer	C25
Prostate cancer	C61
Gallbladder and biliary tract cancer	C23, C24
Breast cancer	C50
Non-Hodgkin lymphoma	C82–C86
Leukemia	C91–95
Multiple myeloma	C90

ICD-10, the 10th revision of the International Classification of Diseases

eTable 2. The Broad-Spectrum Antibiotics Lists

Class	Antibiotic class*	WHO-ATC
Anti-pseudomonal penicillin	Piperacillin	J01CA12
	Piperacillin/tazobactam	J01CR05
	Piperacillin/sulbactam	J01CR05
	Ticarcillin/clavulanate	J01CR03
Anti-pseudomonal cephalosporin	Cefoperazone	J01DD12
	Cefoperazone/sulbactam	J01DD62
	Cefpiramide	J01DD11
	Ceftazidime	J01DD02
	Cefpirome	J01DE01
	Cefepime	J01DE02
Carbapenems	Meropenem	J01DH02
	Imipenem/Cilastatin	J01DH51
	Panipenem	J01DH55
	Ertapenem	J01DH03
	Doripenem	J01DH04
Glycopeptides	Vancomycin	J01XA01
	Teicoplanin	J01XA02

*only included broad-spectrum antibiotics that were available for clinical use in South Korea between 2002 and 2021, based on the national formulary and insurance claims data.
WHO-ATC, World Health Organization's Anatomical Therapeutic Chemical classification system

© 2025 Kim JH et al. *JAMA Network Open*.

eFigure 1. Proportion of Broad-Spectrum Antibiotic Use by Time Period.

This figure illustrates the proportion of patients who received broad-spectrum antibiotics during five end-of-life time intervals for each cancer type: T1 (3–6 months before death), T2 (1–3 months), T3 (2 weeks to 1 month), T4 (1–2 weeks), and T5 (last week of life). (A) composite, (B) anti-pseudomonal penicillin, (C) anti-pseudomonal cephalosporin, (D) carbapenem, (E) glycopeptide.

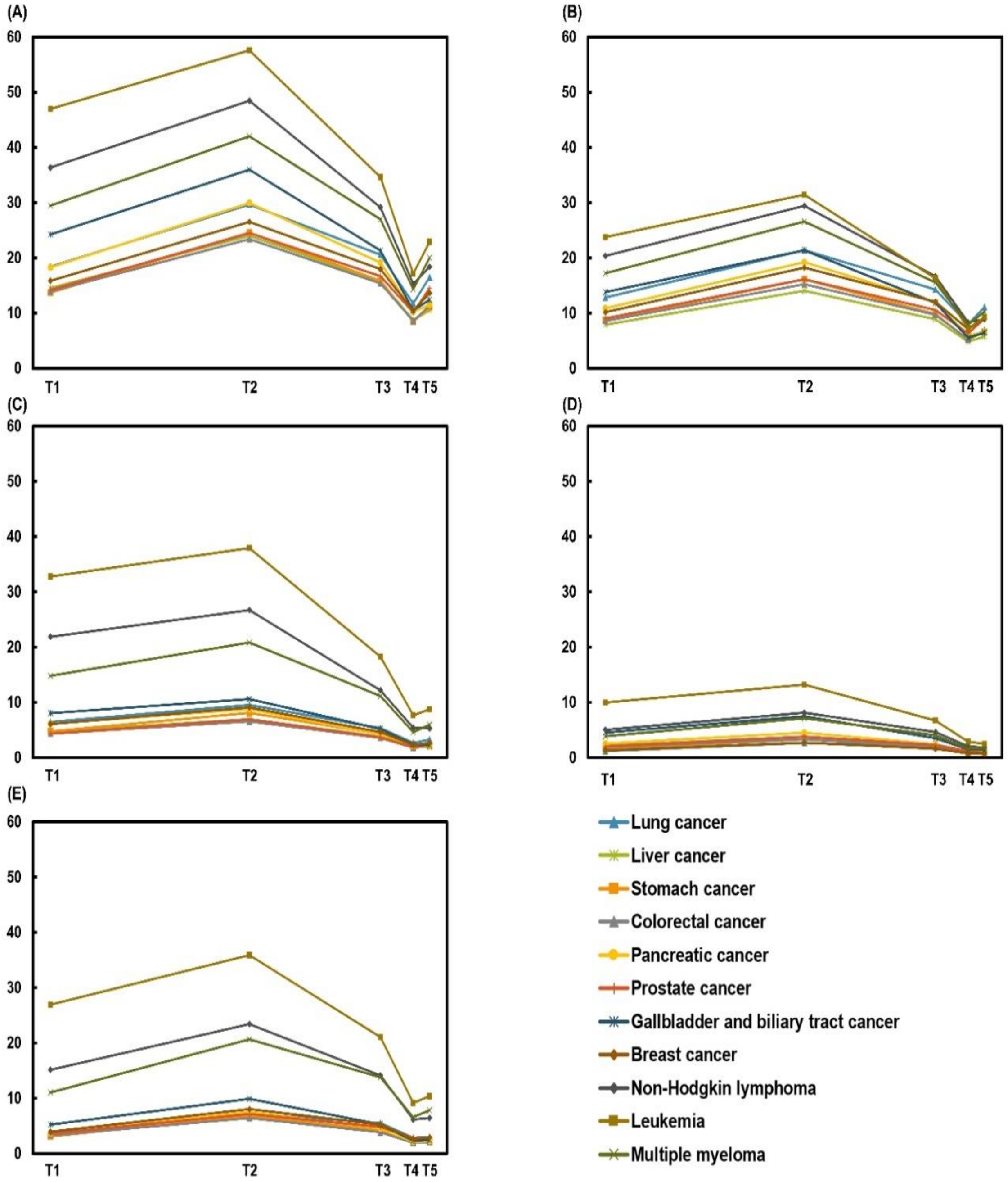

© 2025 Kim JH et al. *JAMA Network Open.*

eFigure 2. Days of Therapy per 1000 Patient-Days of Broad-Spectrum Antibiotic Use by Time Period.

This figure shows days of therapy per 1,000 patient-days across the five end-of-life intervals for each cancer type: T1 (3–6 months before death), T2 (1–3 months), T3 (2 weeks to 1 month), T4 (1–2 weeks), and T5 (last week of life). (A) composite, (B) anti-pseudomonal penicillin, (C) anti-pseudomonal cephalosporin, (D) carbapenem, (E) glycopeptide.

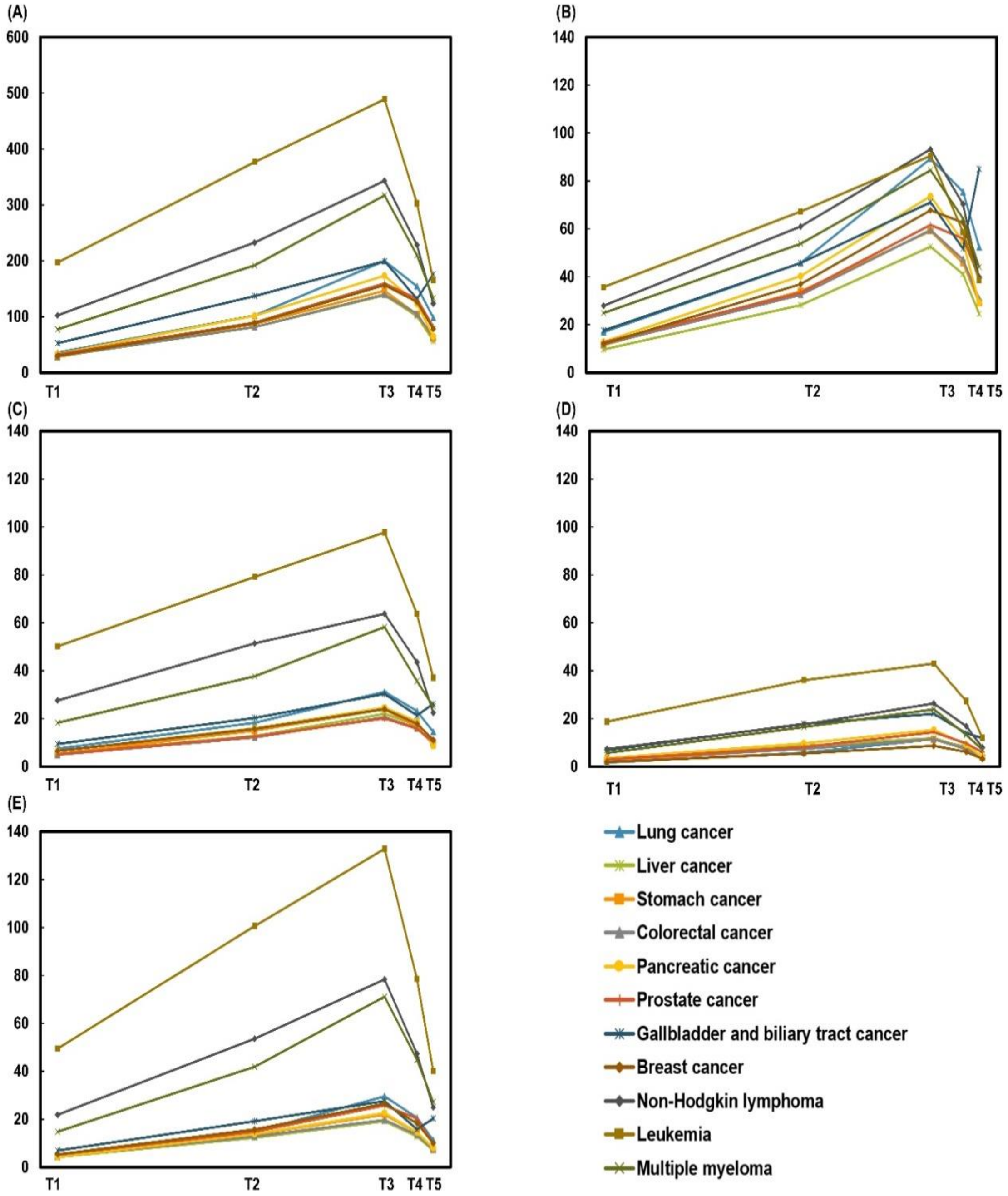

© 2025 Kim JH et al. *JAMA Network Open.*
