# Supplementary material for: Broad-Spectrum Antibiotic Use at the End of Life in Patients With Advanced Cancer
Source: JAMA Netw Open. 2025 Sep 9;8(9):e2530980. doi: 10.1001/jamanetworkopen.2025.30980 (PMC12421337; doi:10.1001/jamanetworkopen.2025.30980)
Supplement: Supplement 2. — Data Sharing Statement [file jamanetwopen-e2530980-s002.pdf]

Data Sharing Statement

Kim. Broad-Spectrum Antibiotic Use at the End of Life in Patients With Advanced Cancer. *JAMA Netw Open*. Published September 09, 2025. doi:10.1001/jamanetworkopen.2025.30980

Data
Data available: No
